# Immediate and persistent efficacy of sarolaner (Simparica™) against *Haemaphysalis elliptica* on dogs

**DOI:** 10.1186/s13071-019-3696-0

**Published:** 2019-09-05

**Authors:** Josephus J. Fourie, Julian E. Liebenberg, Dionne Crafford, Robert Six

**Affiliations:** 1 Clinvet International (Pty) Ltd, Uitzich Road, Bainsvlei, Bloemfontein, South Africa; 2 Zoology Department, University of Johannesburg, Corner of Kingsway and University Roads, Aucklandpark, South Africa; 3 Zoetis Inc., 333 Portage Street, Kalamazoo, Michigan 49007 USA

**Keywords:** Efficacy, *Haemaphysalis elliptica*, Sarolaner, Simparica™

## Abstract

**Background:**

The southern African yellow dog tick, *Haemaphysalis elliptica*, occurs in eastern and southern Africa and adults infest domestic and wild carnivores. This tick species is also a vector of the highly virulent *Babesia rossi* pathogen, the causative agent of canine babesiosis in sub-Saharan Africa. Sustained high levels of efficacy of a parasiticide are not only important in protecting dogs against the direct effects of tick infestation, but also in reducing the risk of tick-borne diseases. Sarolaner (Simparica™ chewable tablets) has been reported to be effective against the major tick species infesting dogs in Europe and the USA, including representatives from the genera *Amblyomma*, *Ixodes*, *Rhipicephalus* and *Dermacentor.* Until now no efficacy evaluations have been reported against species of the genus *Haemaphysalis*. The objective of the study was to confirm the efficacy of a single 2 mg sarolaner/kg oral dose of Simparica™ against induced infestations with *H.* (*R.*) *elliptica*, an important parasite of dogs in southern Africa.

**Methods:**

This blinded, randomised, single centre, placebo controlled efficacy study followed a parallel group design and was conducted on two groups consisting of eight purpose-bred dogs each. Animals were treated orally, once on Day 0, with either a placebo compound (Group 1) or Simparica™ (Group 2). Simparica™ was administered orally at a dose rate of 2 mg sarolaner/kg body weight. The dogs were infested with ticks on Days − 7, − 2, 5, 12, 19, 26 and 33, with removal counts conducted on Days − 5, 2, 7, 14, 21, 28 and 35.

**Results:**

A single oral administration of Simparica™ (sarolaner) at a minimum dose of 2 mg/kg resulted in a 100% efficacy against existing infestations of *H.* (*R.*) *elliptica* on dogs and a 100% reduction in live ticks following weekly re-infestations for 35 days. Moreover, the immediate and persistent high levels of efficacy observed in this study for 35 days is consistent with those observed in previous studies against ticks in other genera.

**Conclusions:**

The efficacy of sarolaner (Simparica™), administered orally to dogs at the minimum label dose of 2.0 mg/kg, was demonstrated against existing and weekly re-infestations of *H*. (*R*.) *elliptica* for at least 5 weeks. Efficacy of 100% was achieved against existing infestations as well as weekly re-infestations.

## Background

Ticks present both a nuisance and a substantial threat to canines, directly and indirectly through the diseases they transmit. Whilst the former pertains to clinical signs of physical damage such as wounds and rashes due to bites, the latter often relates to tick-borne diseases, affecting both wild [1] and domestic [2] dogs.

Prior to 2007, the southern African yellow dog tick, *Haemaphysalis elliptica* (Koch, 1844), was considered a junior synonym or *nomen nudum* of *Haemaphysalis leachi* (Audouin, 1826). However, this changed when Apanaskevich et al. [3] re-described the male and larva of *H. elliptica*, and described the female and nymph for the first time. Adults parasitize domestic and wild carnivores, whilst the immature stages infest rodents [3]. The authors stated that this species is present in southern and eastern Africa and provide numerous records to substantiate their claim. A recent survey in the Limpopo Province of South Africa confirmed the distribution in southern Africa [4]. In comparison, *H. leachi* infests the same host species in Egypt as well as north eastern, central, western and eastern Africa [3]. It follows that previous efficacy studies performed in southern Africa, specifically South Africa, referring to *H. leachi* were in fact conducted against *H. elliptica* [5–7]. However, following the re-description of *H. elliptica*, a number of efficacy studies correctly cite this species [8]. In addition to causing ‘tick worry’ in dogs, *H. elliptica* is a vector of *B. rossi*, the causative organism of canine babesiosis, a common and highly severe disease in these animals in southern Africa [2, 9], proof of the importance of *H. elliptica* to domestic dog health in this African region.

Sarolaner (Simparica™ chewable tablets) is a recently discovered, orally administered, broad-spectrum, isoxazoline ectoparasiticide for dogs [10, 11]. Efficacy of this compound has previously been demonstrated against fleas [12–17], mites [18, 19], ticks [20–27] and ticks and fleas in combination [28, 29]. The efficacy of this active ingredient in preventing transmission of tick-borne diseases has also been evaluated [30]. The latter study demonstrated the efficacy of Simparica™ in preventing the transmission of *Borrelia burgdorferi* and *Anaplasma phagocytophilum* from infected wild-caught *Ixodes scapularis* ticks to dogs.

The percent reductions in mean live tick counts at 1, 2 and 5 days after infestation were 86.3%, 100% and 100% for the group treated with sarolaner 21 days prior to infestation, and 90.9%, 97.1% and 100% for the group treated with sarolaner 28 days prior to infestation. Sustained high levels of efficacy are thus not only important in protecting dogs against the clinical effects of tick infestation, but also important in reducing the risk of tick-borne diseases.

If Simparica™ proves to be equally effective against *H. elliptica*, it may also be an effective tool in preventing the transmission of *B. rossi* by this tick.

Tick species against which Simparica™ have been evaluated include *Amblyomma americanum* [20, 26], *Ixodes scapularis* [21, 26, 27, 29, 30], *Rhipicephalus sanguineus* (*sensu latu*) [22], *Rhipicephalus sanguineus* (*sensu stricto*) [24–26, 28], *Dermacentor reticulatus* [23, 25, 28], *Ixodes hexagonus* [25, 28], *Dermacentor variabilis* [26, 29], *Amblyomma maculatum* [26, 27, 29] and *Ixodes ricinus* [25, 27, 28].

Until now this active ingredient, sarolaner, had not been evaluated against *H. elliptica.* The objective of the study was to confirm the immediate and persistent efficacy of a single 2 mg sarolaner/kg oral dose of sarolaner (Simparica™, Zoetis Inc., Kalamazoo) against induced infestations with *H. elliptica* on dogs.

## Methods

### Animals

Animals used in this investigation were purpose-bred mongrel dogs owned by the test facility (Clinvet). Both placebo (Group 1) and sarolaner (Group 2) treatment groups consisted of two females and six males. All dogs were adult (≥ 6 months). Body weight in Group 1 ranged from 14.4 to 25.2 kg, with an arithmetic average of 19.0 kg. Body weight in Group 2 ranged from 16.60 to 24.00 kg, with an arithmetic average of 19.70 kg. Female dogs were confirmed not to be pregnant or lactating. Each dog was individually identified by electronic transponder (microchip) with a unique and permanent alphanumerical code. Dogs were housed in individual indoor pens such that no physical contact was possible between them, and the possibility of tick transfer among animals was minimal. Dogs were fed an appropriate maintenance ration of a commercial canine diet for the duration of the study. Water was provided in stainless steel bowls and was replenished at least twice daily.

Groups were homogenous, in that body weight did not differ significantly (ANOVA, *F*_(1,15)_ = 0.16, *P* = 0.6947) between groups.

### Design

The study followed a parallel group design and was conducted on two groups of eight purpose-bred dogs each, selected from an enrolled group of 20 dogs. All the enrolled animals underwent a 14-day acclimatisation period, from study day (Day) − 14 to Day − 1. Clinical examinations were performed on Day − 14.

Allocation of animals to groups (performed on Day − 12) and administration of the investigational veterinary product (IVP) and placebo (performed on Day 0) were the responsibility of a non-blinded person. Body weight and hair length were measured at the same time. After allocation, non-blinded personnel were not actively involved in any other experimental procedures, except those mentioned above. All other people involved in the study were blinded to the allocation of groups.

The 16 dogs included in the study were ranked within sex in descending order of individual tick counts, performed prior to administration of the placebo and IVP. After ranking, animal identification numbers (IDs) were allocated to eight blocks consisting of two dogs each. Animal IDs were used to break ties. Within blocks, dogs were randomly allocated to two coded groups. To achieve blinding, Group 1 and Group 2 were randomly assigned to the treatment groups by a non-blinded person. Blinding codes were only revealed after completion of the animal phase of the study.

Tick infestations were performed on Days − 7, − 2, 5, 12, 19, 26 and 33, with removal counts performed on Days − 5, 2, 7, 14, 21, 28 and 35. Animals in Group 1 were treated orally with a placebo compound, whilst the IVP was administered orally to animals in Group 2. Both products were administered once only on Day 0 of the study. The dogs were observed prior to treatment, and thereafter at 1, 3, 6 and 24 h after treatment for possible adverse events. Health observations were performed twice daily from Day − 14 to Day 35. Throughout this period daily minimum and maximum temperatures were measured in the cage environment, which ranged from 17.8 to 26.9 °C. The relative humidity was also measured daily in the cage environment, and ranged from 32.8 to 65.2%.

### Investigational veterinary product (IVP) and placebo description and dose rate

Both the IVP and placebo were flavoured, hard, chewable formulations intended for oral administration. The IVP chewable tablets (Simparica™) contained sarolaner as the active ingredient, whilst the placebo chew tablets contained only the vehicle and no active ingredients.

Tablet sizes available were 5, 10, 20, 40 and 80 mg. The target dose rate was 2 mg sarolaner per kg body weight. A dosage table was compiled, listing animal weight ranges and the combination of tablets to be administered to animals within each weight range, in order to get as close as possible to the target dose. The same dose table was used for both the IVP and placebo formulations.

### Administration of the investigational veterinary product (IVP) and placebo and related procedures

Food was withheld overnight prior to administration of the IVP and placebo. Each dog was offered its regular food ration 20 min (± 10 min) before administration of the IVP and placebo. The food of each dog was weighed prior to feeding (‘weight offered’), and was weighed again 20 min (± 10 min) after dosing (‘weight remaining’). Any remaining food was offered *ad libitum*.

Immediately before administering the IVP or placebo to an animal, the designated person confirmed the animal’s identification. Day − 2 body weights in the dose table were used to calculate the dose to be administered to each dog. During IVP and placebo tablet administration, disposable gloves and aprons were worn and were changed between animals of the different groups. The table on which each dog was placed, was cleaned with a disinfectant between animals.

The tablet(s) were administered orally, and swallowing was encouraged by simultaneously administering approximately 10 ml of water by means of a syringe. Each dog was observed for several minutes after dosing to ensure that the tablets were swallowed, and for potential adverse events associated with the administration of a whole chewable tablet. No incomplete dosing was recorded.

Doses were targeted to meet the proposed minimum oral dose of 2.0 mg/kg. For the sarolaner group, the actual dose rate range was 2.0 to 2.3 mg/kg.

### Ticks

Adult ticks from a laboratory-bred strain of *H. elliptica* (South African origin) were used for artificial infestation. Ticks used for infestation were unfed, at least 1 week old (mean age 4.5 months at the start of the study, same batch used for both groups) and of a balanced sex ratio (50% female: 50% male). The *H. elliptica* strain used was collected in February 2012 from dogs in the rural surrounds of Bloemfontein, Free State Province, South Africa. Since collection, the strain has not been enriched with other collected or laboratory strain ticks, and has been maintained on donor animals at Clinvet International (Pty) Ltd. for three cycles prior to being used in this study.

### Tick infestations and assessment

Each dog was artificially infested with 50 ticks on each of the infestation days as described above. The time of infestation was recorded for all animals. Counting and removal of ticks were as close as possible to the specified target times (48 ± 2 h after infestation or after placebo or IVP administration).

Ticks were detected by palpation and by direct observation following parting of the hair and collected. Each dog was also combed thereafter to ensure that all ticks were removed and counted. Body regions examined, not necessarily in this order, were: outer surface of hind legs, including feet; tail and anal areas; lateral areas, not including shoulders; abdominal area, from chest to inside hind legs; fore legs and shoulders, including feet; all neck and head areas; and dorsal strip from shoulder blades to base of tail.

The ticks collected from each dog were recorded by sex according to the survival and attachment status described by Marchiondo et al. [31] (Table 1), as the more recent Marchiondo et al. [5] reference was not available at time of protocol preparation.

**Table 1:** Survival and attachment status categories of ticks during assessments

Category	General findings	Attachment status
1	Live	Free
2	Live	Attached; unengorged^a^
3	Live	Attached; engorged^b^
4	Killed	Free
5	Killed	Attached; unengorged^a^
6	Killed	Attached; engorged^b^

^a^No filling of the alloscutum evident

^b^Obvious or conspicuous filling of the alloscutum evident

### Statistical analysis

Efficacy was calculated for the IVP group at each assessment day according to the formula below. Efficacy calculations were based on arithmetic means, but geometric means were also presented and analysed as supportive evidence.

Geometric means were calculated using the tick data (count + 1) and one (1) was subsequently subtracted from the result to obtain the geometric mean tick counts for each group.

$${\text{Efficacy}}\;(\% ) = 100 \times \left( {{\text{M}}_{\text{c}} - {\text{M}}_{\text{t}} } \right)/{\text{M}}_{\text{c}}$$

where Mc is the arithmetic or geometric mean number of live ticks (categories 1 to 3) on dogs in the placebo control group (Group 1) at a specific time point, Mt is the arithmetic or geometric mean number of live ticks (categories 1 to 3) for immediate (Day 2) and persistent (Days 7, 14, 21, 28 and 35) efficacy calculations on dogs in the IVP treated group (Group 2).

As a primary comparison, the groups were compared using an analysis of variance (ANOVA) (Proc GLM procedure in SAS) with an administration effect on untransformed tick count data. The groups were also compared using an ANOVA (Proc GLM procedure in SAS) with an administration effect after a logarithmic transformation on the tick (count + 1) data.

SAS v.9.3 TS Level 1M2 was used for all the statistical analyses. The level of significance of the formal tests was set at 5%; all tests were two-sided.

The IVP was considered effective if the adulticidal efficacy was ≥ 90% against ticks, and adequacy of infection had been demonstrated in the control group.

## Results

### Health observations

Abnormal clinical signs observed during the clinical examination on Day − 14 included enlarged lymph nodes in a single animal in the placebo group (287 68B) and in the IVP group (B8B 8B2). One animal in the placebo group (CC5 CDA) also presented with a permanent scar. All the dogs were judged fit for inclusion by the attending veterinarian.

During the daily observations, one dog (CC5 CDA, placebo group) presented with superficial dermatitis on Day 22, while dog DF4 CE1 (IVP group) presented with a broken toenail during the 1 h specific post-administration observation. Neither of these conditions were considered serious, and the animals received concomitant therapy under supervision of the examining veterinarian.

None of the dogs vomited following dosing, but one dog (E9C 269) in the IVP group momentarily gagged immediately following the administration of water. The animal recovered without any further adverse reactions.

None of the clinical signs observed during the study were thought to be related to administration of the IVP or placebo.

### Evaluation of efficacy

The arithmetic and geometric mean numbers of *H. elliptica*, and efficacies based on these, calculated for the various days of assessment, are summarized in Table 2.

**Table 2:** Arithmetic and geometric mean tick^a^ counts and efficacy of the IVP^b^

Day	Placebo^c^		Sarolaner		% efficacy
	Mean^d^	Range	Mean	Range	
2	26.8 (27.9)	18–49	0	0–0	100
7	31.9 (32.5)	22–40	0	0–0	100
14	36.8 (37.4)	30–48	0	0–0	100
21	26.2 (27.3)	14–35	0	0–0	100
28	30.6 (31.4)	19–41	0	0–0	100
35	39.2 (39.9)	26–49	0	0–0	100

^a^Laboratory tick strain used was obtained from Clinvet, Bloemfontein, Free State, South Africa

^b^Commercial chewable tablet formulation (Simparica™) containing sarolaner as active ingredient

^c^Chewable tablet with vehicle compounds only

^d^Mean values indicated as: geometric (arithmetic)

*Note*: Difference in mean tick count was significant (ANOVA, *F*_(1, 15)_ = 69.41, *P* < 0.0001) in all instances) for all assessment days

The arithmetic mean tick counts on the dogs in the placebo treatment group ranged from 27.3 to 39.9, indicating a vigorous tick challenge on all assessment days. Adequacy of infestation had thus been achieved.

No ticks were present on animals in the sarolaner treatment group at any time point post-treatment, resulting in 100% efficacy against tick challenges with adult *H. elliptica* for the entire duration of the five-week study period.

## Discussion

Until recently, pet owners and practitioners had to rely on topical acaricidal treatments for the treatment and control of ticks on dogs. The recent development of oral formulations in the isoxazoline class of compounds, which had proved to be highly effective acaricides, provided an attractive alternative mode of treatment to topically administered products [10, 11]. Sarolaner, a new member of the isoxazoline class, has proved to be effective against the main tick species that infest dogs in countries both within the European Union (EU) and in the USA. Although these studies demonstrated the efficacy of sarolaner against tick species in the genera *Ixodes* [21, 25–30], *Amblyomma* [20, 26, 27, 29], *Rhipicephalus* [22, 24–26, 28] and *Dermacentor* [23, 25, 26, 28, 29], to our knowledge no study to ascertain the efficacy against ticks from the genus *Haemaphysalis* has been reported.

In the present study, a single treatment with sarolaner (Simparica™) provided a 100% efficacy against existing *H. elliptica* infestations as well as a 100% reduction in live ticks following weekly re-infestations for 35 days. Thus, the claimed one-month efficacy of sarolaner, administered at a minimum dose of 2 mg/kg, against other tick species, was verified against *H. elliptica*, an important species in the genus *Haemaphysalis* that infests dogs. Moreover, the immediate (Day 2) and persistent (Days 7, 14, 21, 28 and 35) high levels of efficacy observed for 35 days in this study is consistent with that observed for other ticks in the genera *Ixodes*, *Amblyomma*, *Rhipicephalus* and *Dermacentor* that commonly infest dogs [20–30].

Sustained high levels of efficacy over the entire treatment period is not only important in protecting dogs against the clinical effects of tick infestation, but also reduces the risk of tick-borne diseases. In the case of *H. elliptica*, the specific pathogen of interest is *B. rossi* [9]. The time of transmission of *Babesia* species has not yet been defined; however, the minimal time to transmission is estimated from 36 hours after tick attachment onwards [32]. Moreover, for complete protection, high levels of efficacy must not only be sustained, but also not be affected by external factors such as rain and bathing. Since the adults of *H. elliptica* most commonly infest dogs with an outdoor lifestyle [1, 4], the highly efficacious, systemically active sarolaner oral chewable formulation (Simparica™) is an attractive treatment option for dog owners and practitioners.

Furthermore, the benefit of sustained activity may significantly reduce the risk of transmission of *B. rossi*, one of the most virulent piroplasms infecting dogs, by *H. elliptica*. [1, 2, 9].

## Conclusions

The efficacy of sarolaner (Simparica™ chewable tablet), administered at the proposed minimum oral dose of 2.0 mg/kg, was demonstrated against existing and weekly re-infestations of *H. elliptica* for at least 5 weeks. Efficacy of 100% was achieved against existing infestations as well weekly re-infestations for 35 days.

## Data Availability

Study documentation is archived at the test facility (Clinvet) and sponsor localities, but is not freely available because of confidentiality agreements.

## Abbreviations

ANOVA: analysis of variance
Day: study day
GCP: good clinical practice
IVP: investigational veterinary product
VICH: Veterinary International Committee on Harmonisation (international cooperation on harmonisation of technical requirements for registration of veterinary medicinal products)
WAAVP: World Association for the Advancement of Veterinary Parasitology
